# Molecular Cascades That Build and Connect Auditory Neurons from Hair Cells to the Auditory Cortex

**Published:** 2025

**Authors:** Ebenezer N. Yamoah, Gabriela Pavlinkova, Bernd Fritzsch

**Affiliations:** 1 Department of Translational Neurosciences, University of Arizona, College of Medicine, Phoenix, AZ, 85004, USA; 2 Laboratory of Molecular Pathogenetics, Institute of Biotechnology CAS, Prumyslova 595, 25250 Vestec, Czechia; 3 Department of Neurological Sciences, University of Nebraska Medical Center, Omaha, NE, 68198, USA

**Keywords:** Spiral ganglion neurons, Cochlear nuclei, Cochlear hair cells, Brainstem, Genetic basis, Auditory cortex

## Abstract

Understanding the development of the auditory system is crucial for uncovering the molecular origins of hearing and its related disorders. During this development, spiral ganglion neurons extend peripheral fibers to cochlear hair cells and central projections to the cochlear nuclei, setting up a tonotopic map that connects the ear to the brainstem, enabling frequency-specific sound perception. This sensory information is then integrated bilaterally through a relay involving the superior olivary complex, lateral lemniscus, inferior colliculus, medial geniculate body, and the auditory cortex. While anatomical connectivity has been well-documented, recent advancements have revealed gene regulatory networks that coordinate the specification, differentiation, and connectivity of auditory neurons. In this review, we examine the molecular cascades guiding auditory system development, emphasizing transcriptional hierarchies and lineage determinants. Insights into these mechanisms enhance our understanding of auditory circuit formation and provide a critical foundation for therapeutic strategies aimed at addressing congenital and acquired hearing loss.

## Introduction

Mammals are the only vertebrates known for their ability to hear high frequencies above 10 kHz. They possess a unique organization of cochlear hair cells (HCs), which are divided into inner and outer hair cells (IHCs and OHCs) that receive innervation from spiral ganglion neurons (SGNs). SGNs transmit auditory signals to the cochlear nuclei (CNs), which relay the information to higher auditory processing centers, including the auditory forebrain. This overview addresses the genetic mechanisms that establish tonotopic organization in the auditory system: IHCs connect the brain to SGNs, which are organized in the cochlea in a frequency-specific manner, with high frequencies at the base and low frequencies at the apex. SGNs project centrally to CN neurons, which are also tonotopically organized, preserving the frequency map from the periphery to the brainstem. Reciprocal connections ease the encoding, transmission, and interpretation of auditory information. Recent studies have enhanced our understanding of the genetic basis for the development of the auditory system and the molecular processes that shape hearing function, paving the way for novel therapeutic strategies that may alleviate hearing loss in children and older adults [1,2]. Recent gene therapy clinical trials suggest that targeting specific genetic mutations, such as *TMC2* and *OTOF*, could enable the restoration of hearing in affected individuals [3,4].

The review will outline the genetic basis for the cochlea, brainstem, midbrain, and cortex, which define the molecular basis for hearing. We try to integrate specific mechanistic overlaps that present more recent approaches to guide future research detailing the effect of central projections without tonotopic organization. We provide literature citations of recent papers that may inform future research directions in cochlear and central nervous system development and guide therapeutic approaches.

### Building Spiral Ganglion Neurons to Connect the Cochlear Nuclei with Hair Cells

**Figure 1.**
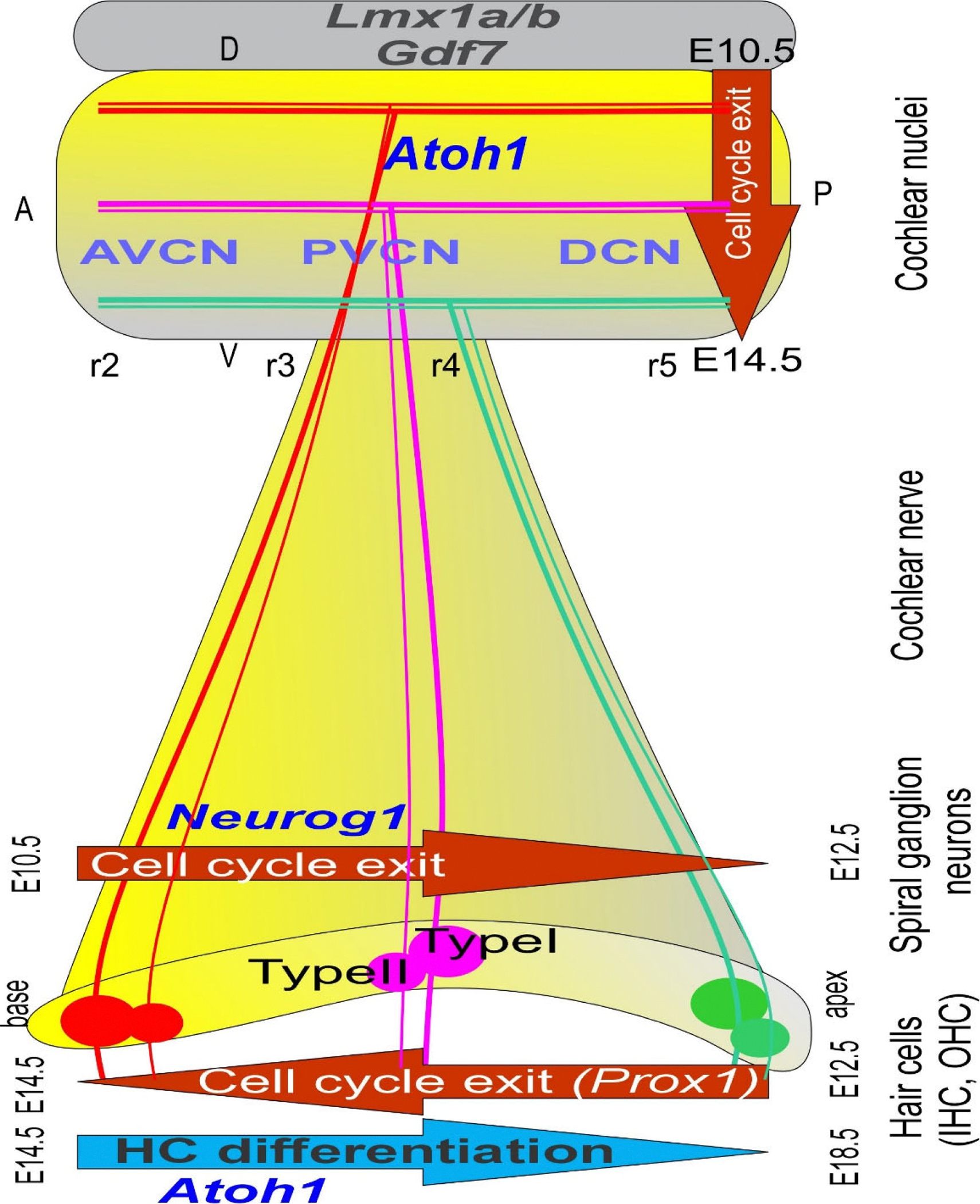
The proliferation of hair cells, spiral ganglion neurons, and cochlear nuclei is displayed. *Neurog1* is expressed first and progresses from the base to the apex. Proliferation overlaps with cochlear nuclei from dorsal to more ventral regions. The proliferation of hair cells begins in the apex (*Prox1*) but upregulates later (*Atoh1*) to reach the apex much later. The first connection originates from the basal turn to innervate the dorsal part of the cochlear nuclei, showing a delay of inner hair cell development, while the apex extends to the ventral cochlear nuclei, demonstrating a significantly later progression of apical hair cell formation. Two types of fibers connect the hair cells and the cochlear nuclei: the thinner type II fibers. AVCN: Anteroventral Cochlear Nuclei; *Atoh1*: Atonal homolog 1; DCN: Dorsal Cochlear Nuclei; Gdf7: Growth differentiation factor 7; *Neurog1*: Neurogenin 1; Lmx1a/b: LIM homeobox transcription factor 1 alpha/1 beta; *Prox1*: Prospero homeobox 1; PVCN: Posteroventral Cochlear Nuclei. Modified from [68].

Molecular and cellular processes govern the development, differentiation, and maintenance of cochlear hair cells (HCs), spiral ganglion neurons (SGNs), and cochlear nuclei (CNs). Understanding the recurring roles of key transcription factors requires an integrated approach, including *in situ* hybridization and immunohistochemistry, as well as gene knockout mouse models, to reveal their spatiotemporal regulation during auditory pathway development. HCs, SGNs, and CNs differentiate as distinct but temporally overlapping hubs. SGNs proliferate and develop in a base-to-apex gradient from embryonic day E10.5 to E12.5. In contrast, while HC cell cycle progression follows the opposite direction, from the apex (E12.5) to the base (E13.5), HC differentiation initiates near the cochlear base around E13.5 and continues toward the apex until E18.5 [5]. CN neurons proliferate between approximately E10 and E14, overlapping with SGNs and HCs. SGNs and HCs are derived from the ear placode, while CN neurons are derived from rhombomeres (r2-r5) from the dorsal brainstem [6]. SGN fibers reach the CN at E12 at the basal turn, whereas the topology between the basal and apical turn afferents is established at E14.5 (Figure 1). SGN fibers reach IHCs via type I fibers starting at E15 and then OHCs via type II fibers at E18 [7]. While SGNs send input to the CN, the fibers project to several subnuclei, the anteroventral cochlear nucleus (AVCN), posteroventral cochlear nucleus (PVCN), and dorsal cochlear nucleus (DCN; Figure 1), each with distinct anatomical and functional characteristics important for frequency tuning, intensity coding, and temporal processing [8].

### SGNs depend on *Eya1/Smarca4*, *Sox2,* and *Neurog1*

Upstream, the HDACs are responsible for neuronal differentiation, shedding light on HDAC and REST inhibitors as potential targets for use as therapeutic agents for the treatment of neurodegenerative diseases [9,10]. *Eya1/Six1/Smarca4* and *Sox2* define the neurosensory domain of the inner ear precursors, whereas *Neurog1* initiates neuronal development [11,12]. A unique trio of transcription factors, *Tbx1–3*, in concert with *Neurog1,* regulates otocyst neurogenesis [13]. The transcriptional repressor *Tbx1* acts as a selective gene, controlling neuronal fate in the otocyst, while deletions of both *Tbx2* and *Tbx3* expand the otic neurogenic domain and disrupt inner ear morphogenesis. Conditional deletions of *Gata3, Lmx1a/b, Dicer,* or *Shh* result in a complete loss of SGNs [14–16], while nearby developing vestibular neurons form. Deletions of the transcription factors *Neurod1* and *Isl1* result in abnormal migration of SGNs [17–19]. Neurotrophins are needed for SGN viability and maturation [20], with the loss of all SGNs occurring in the absence of the neurotrophins encoded by *Bdnf* and *Ntf3* or the genes encoding their receptors, *TrkB* and *TrkC* [21]. A possible downstream effect provides evidence for a pathophysiological mechanism of deafness and shows how genes involved in different forms of deafness may interact together, including *Bdnf/Ntf3* [22,23].

Type I SGNs can be classified into three major subtypes: type Ia-c [24]. Type Ia SGNs are characterized by enriched expression of *Calb2* and *Pcdh20,* which later becomes *Runx1-*positive [25]. Type Ib SGNs show enriched expression of *Calb1,* while type Ic SGNs show expression of *Pou4f1*. Type Ia afferents project preferentially to the pillar aspects of the IHCs [24,26] and correspond to a high spontaneous rate, whereas type Ic SGNs project preferentially to the modiolar face of the IHCs and correspond to low spontaneous rate fibers. Type Ia, Ib, and Ic SGNs show subtle variations in their distribution along the apical, middle, and basal turns of the cochlea. Approximately 7% of SGN afferents form type II neurons that are selectively *Prph-*positive at a specific developmental stage. The type II neurons innervate OHCs and also reach out to the CN [7]. All hair cells depend on Otoferlin (*Otof*), which is needed for glutamate release. Without Otoferlin, the proper activation of the SGNs or CNs is profoundly disrupted [27,28]. Indeed, human mutations of *OTOF* can be rescued by inserting the mutant gene with the normal *OTOF* form and restoring hearing [3].

### HCs depend on *Eya1/Smarca4*, *Sox2,* and *Atoh1*

Like SGNs, HC progenitors initiate development through the activity of transcriptional regulators *Eya1, Six1, Hdac’s, Rest* and *Smarca* [10,11]. Downstream expression of the transcription factor *Sox2* is required to initiate HC formation [12]. *Atoh1* is obligatory for further differentiation of HCs [29]. Downstream genes include the transcriptional regulators (*Pou4f3, Gfi1,* and *Barhl1*), which are necessary for HC maintenance [30]. Deletions of the transcription factors *Gata3, Lmx1a/b*, or *Pax2* result in the absence of all HCs [14,15,31]. Loss of the *bHLH* transcription factor *Neurog1*, and other regulatory factors such as *Lmx1a* or *Foxg1,* disrupts *Atoh1* expression and impairs HC differentiation, resulting in a shortened cochlea with multiple rows of HCs and supporting cells, or a fate switch from cochlear to vestibular-like HCs, as shown in *Foxg1*, *Neurog1,* and *Lmx1a* mutants [29,32,33]. *Irx3/5* DKO mice show a fusion of the basal cochlear turn with the saccule, the development of vestibular-like HCs with an incomplete segregation of topological projections to the cochlear nuclei [34]. A shorter cochlea and conversions of OHCs into IHCs are associated with the deletion of *Neurod1* and *Isl1* [18,35]. *Tbr2* (aka *Eomes*) regulates the cycling of neurons and hair cells by interacting with *Dll*, *Notch* and *Hes* [36,37]. The differentiation between IHCs and OHCs depends on distinct genetic programs. OHC development requires downstream expression of the transcriptional repressor *Insm1* and transcriptional regulator *Ikzf2* [38]. Loss of the transcriptional repressor *Tbx2* converts IHC into OHC-like HCs; a similar transformation is documented with *Bcl11a* deletion [39]. In contrast, IHCs do not develop without *Srrm3/4* [40]. Many more null mutations are presented in recent papers that detail the null deletion of *Meis2*. Results suggest that several additional gene expressions may be required to develop new hair cells [41,42].

### CN neurons depend on *Atoh1* and *Ptf1a*

While SGNs and HCs show limited variability, CN neuron populations arise from distinct progenitor domains, notably *Atoh1* and *Ptf1a,* that generate dA1 (*Atoh1*) and dA4/dB1 (*Ptf1a*) neurons. Spherical bushy cells form a large calyx in r2, while globular bushy cells interact with significant gaps of the calyx and belong r3 [43,44]. D-, L-, and T-stellate cells likely derive from r3 [8], with T-stellate cells being glutamatergic and D-stellate cells glycinergic. The r4 neuronal populations are represented by octopus cells (PVCN) and giant cells, which are dependent on *Atoh1* expression [45]. The r5 populations include *Atoh1*-positive-derived neurons (unipolar brush cells, fusiform cells, and granule cells) and a large added subpopulation of *Ptf1a*^*+*^*-positive*-derived neurons (GABAergic Golgi cells, superficial stellate cells, glycinergic cartwheel, and tuberculoventral cells). Type I SGNs connect with *Atoh1-*positive-derived neurons in the CN as end bulbs or end in small connections within the topological organization. The type I and type II fibers are parallel, but type II fibers only reach the granule cells [46]. All glutamate neurons in the CN derive from the *Atoh1-positive* domain (spherical, globular, T-stellate, roof, octopus, fusiform, giant, unipolar brush cells (UBCs), and granule cells). Neurons that are derived from the *Ptf1a-*positive domain are either glycinergic (possibly from dA4; D-stellate, cartwheel, and tuberculoventral cells) or GABAergic (possibly from dB1; Golgi, superficial stellate cells [6]). Moreover, *Otof* is required for the proper activation of cochlear hair cells and cochlear nuclei to allow functional interaction [47].

**Figure 2.**
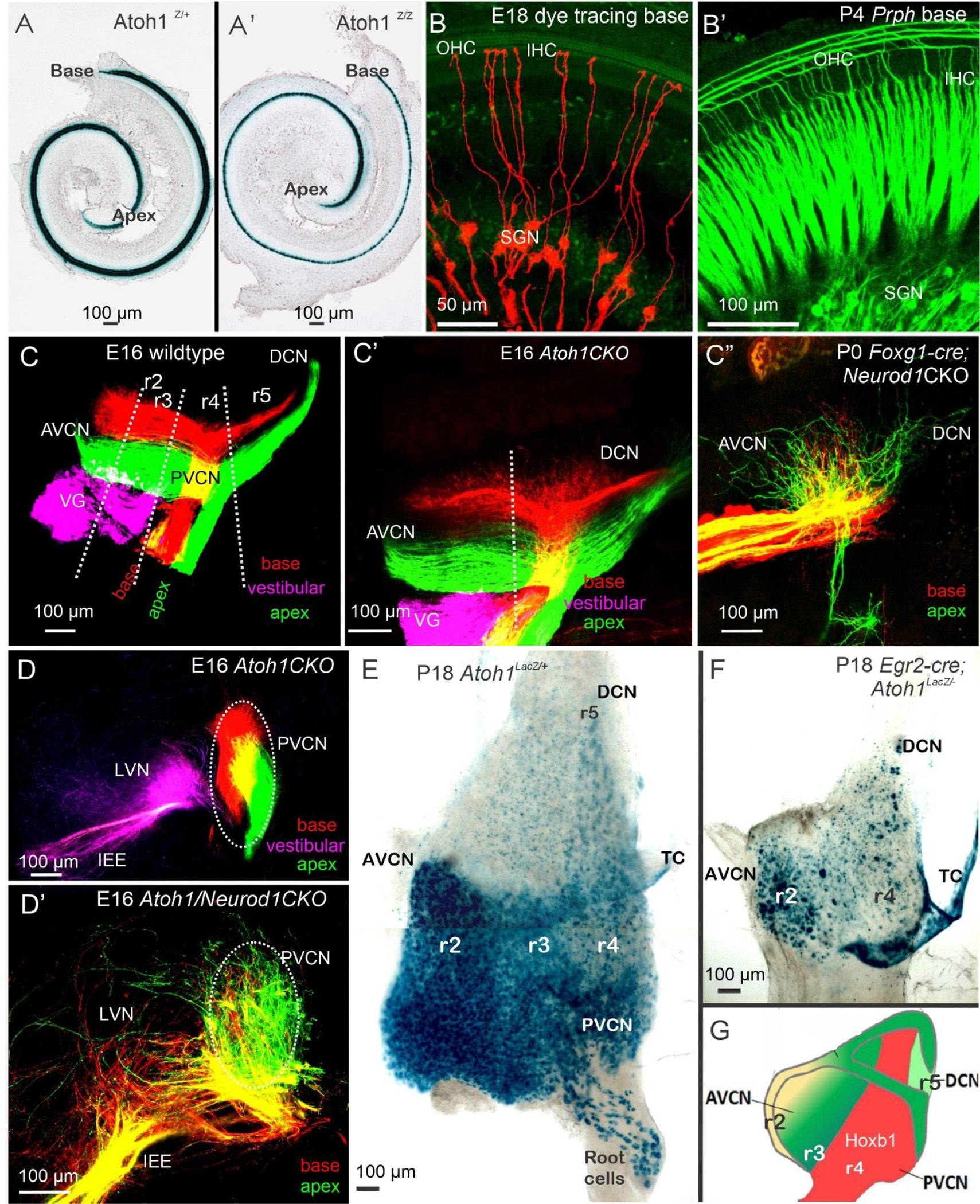
Development of SGNs, HCs, and central projection. All hair cells depend on *Atoh1* (**A**), which initially develop even without *Atoh1* expression (**A’**). Applying dye tracing selectively labels the SGNs that innervate the IHC (**B**). Prph expression indicates the innervation of OHCs from type II fibers (**B’**). The central projection displays the tonotopic organization in control mice (**C**). Even in the presence of *Atoh1*, fibers can develop to innervate the cochlear nuclei (**C’**). *Neurod1* deletions show overlapping fibers that innervate the small CN (**C”**). Sections reveal segregation in *Atoh1* null mice (**D**), while the absence of *Atoh1* and *Neurod1* results in overlap (**D’**). A flat mount of the CN using *Atoh1*LacZ indicates the four rhombomeres with a gradient of *Atoh1* (**E**). Combining Atoh1-Lacz with genetic engineering using *Egr2*-cre (also known as Krox20) demonstrates the absence of r3 and r5, and a reduced generation of r2 and r4 (**F**). In red, the Hoxb1-positive neurons generate r4 neurons. Abbreviations: *Atoh1*: Atonal homolog 1; AVCN: Anteroventral Cochlear Nuclei; DCN: Dorsal Cochlear Nuclei; *Egr2*: Early growth response 2; HCs: Hair Cells; IHC: Inner Hair Cells; *Neurod1*: Neurogenic differentiation 1; OHC: Outer Hair Cells; *Prph*: Peripherin; PVCN: Posteroventral Cochlear Nuclei; r2-r5: Rhombomere 2- rhombomere 5; SGNs: Spiral Ganglion Neurons; TC: Tela Choroidea; VG: Vestibular Ganglion Neurons. Taken from [2,35,44,50,69].

Loss of *Ptf1a* results in the overproduction of neurons from the *Lmx1b* lineage (Figure 1), rather than *Lhx1/5* and *Pax2* expression in dB1 neurons [6,48]. Conversion shows a graded effect that turns r2–3 into *Lmx1b*-positive neurons instead of dB1, while r4–6 transforms from dA4 into dA3 in the *Ptf1a*^*−/−*^. Utilizing either *Egr2-cre* or *Hoxb1-cre* [40] corresponds with the origin of dA1 and dA4/dB1-derived neurons [48]. *Atoh1* deletion causes the loss of r3- and r5-originated neurons and nearly complete elimination of the AVCN in *Egr2*-cre; *Atoh*1CKO (conditional knockout) (Figure 2), while most r4 neurons from the PVCN and DCN are lost in *Hoxb1-cre; Atoh1CKO*. Deletion of *Bhlhb5* removes two distinct types of DCN neurons: unipolar brush cells and cartwheel cells. Furthermore, *Lbx1* is expressed in stellate and cartwheel cells of the DCN, but not in Golgi cells, implying a diversification of genetic programs among distinct types of inhibitory neurons, specifically *Ptf1a*-lineage neurons [6]. More recent analysis has found distinct neuronal populations with unique gene expression [8] that require further investigation to connect their origin (*Atoh1; Ptf1a*) with their adult expression. The absence of *Neurod1* or *Isl1* results in disorganized SGN fibers that connect with reduced input, allowing for an unusual tonotopic organization [18,19]. The lack of *Ptf1a* likely results in the absence of inhibitory GABAergic neurons, which can disrupt normal function by disrupting Cl^−^ homeostasis [49].

**Figure 3.**
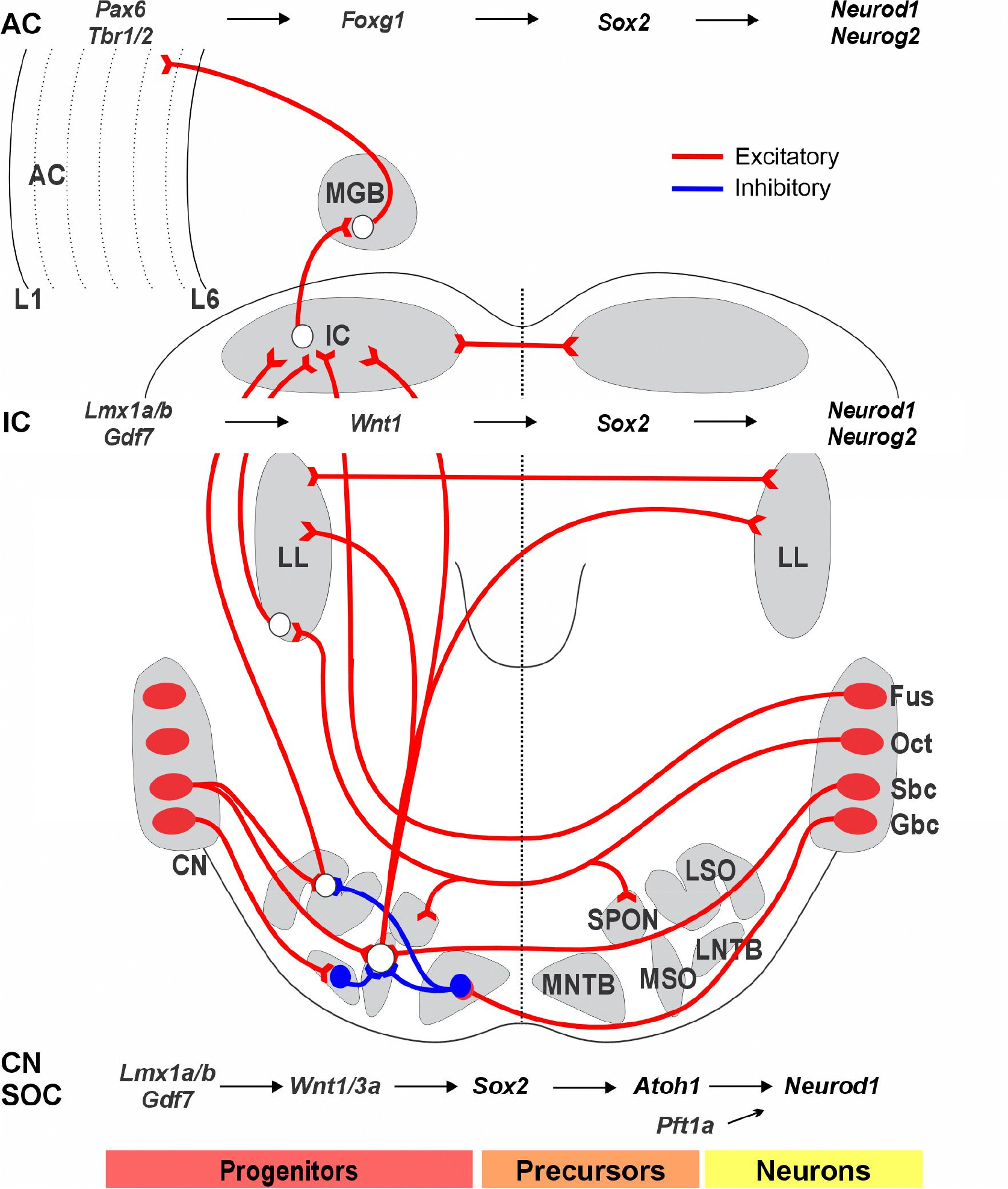
The output of neurons in the cochlear nucleus (CN) projects to the superior olivary complex (SOC). CN/SOC depend on *Lmx1a/b*, *Gdf7*, and *Wnt1/3a*, which guide the migration of SOC from r4. Bilateral interactions involve the lateral superior olive (LSO), medial superior olive (MSO), medial nucleus of the trapezoid body (MNTB), and lateral nucleus of the trapezoid body (LNTB): LSO receives binaural input from the ipsilateral spherical bushy cells (SBCs) and contralateral globular bushy cells (GBCs), which is inhibited by neurons from the MNTB. MSO receives bilateral excitatory input from both ipsilateral and contralateral SBCs and inhibitory input from GBCs via the MNTB and LNTB. The octopus cells (OCT) provide bilateral input to the superior paraolivary nucleus (SPON) and connect with the lateral lemniscus (LL). The dorsal cochlear nucleus, originating from fusiform neurons, bypasses the SOC to reach the contralateral inferior colliculus (IC). Progenitors in the IC depend on *Lmx1a/b*, *Gf7*, *Wnt1*, and *Sox2* to generate neurons, which then rely on *Neurod1* and *Neurog2* to differentiate into excitatory glutamatergic neurons. Additionally, the medial geniculate body (MGB) receives input from the IC and projects to the auditory cortex (AC). Progenitors in the auditory cortex depend on *Pax6*, *Tbr1/2*, and *Foxg1* to induce *Sox2* to create neurons that depend on *Neurod1* and *Neurog2* to differentiate into excitatory glutamatergic neurons. These interactions play significant roles in processing auditory information along the neural pathway. The molecular factors guiding the development of these projection pathways are poorly understood. Images are compiled from various sources. Modified after [2].

Overall, SGNs rely on *Neurog1*, while cochlear HCs and CN neurons depend on *Atoh1*, with *Ptf1a* also being involved in the CN (Figure 3). The cochlear system evolves from a relatively simple gene expression program into a specialized structure, consisting of only four neuronal subtypes that innervate just two types of HCs. This contrasts with the greater neuronal diversity of the CN, which includes at least 12 distinct neuron types. Each input is segregated and overlaps with distinct projections that coordinate functional expression across various gene families, thereby customizing projection patterns. Note that the central projection is tonotopic, receiving fibers from the base, middle, and apex to provide frequency-specific input.

### Transformation of Unilateral Sound Input into Bilateral Auditory Information through SOC, IC, MGB, and AC Interactions

The superior olivary complex (SOC) receives auditory information from the CN and serves as the foundation for binaural auditory processing (Figure 3). Neurons destined for the SOC are generated between embryonic day 10.5 (E10.5) and embryonic day 17.5 (E17.5). During this time, they primarily migrate, differentiate, and organize to form the SOC [50]. The SOC neurons are partially dependent on *Atoh1* and the homeobox-containing gene *En1* [51], with a sizable number originating in r4 and 5. An incomplete deletion of rhombomeres 3/5 using *Krox20 (Egr2)-cre* deletion of *Atoh1* reveals a small remaining population with reduced SOC input [44]. It stays unclear how many SOC neurons are lost directly in the absence of the CN.

The inferior colliculus (IC) serves as a key integration center for auditory input, receiving signals from both the CN and SOC and directing its output to the medial geniculate body (MGB). Various genes play crucial roles in the development of the IC [52]. The gene *Opb* encodes a small GTPase of the Ras superfamily that antagonizes *Shh*, a vital signaling molecule involved in the early patterning of the IC [52]. A complete loss of *Wnt1* acts downstream of *Lmx1b*, leading to the elimination of the entire midbrain (Figure 3). Deletions of *Fgf8* and *Pax2*, among others, reduce the volume of the IC [53,54]. In contrast, overexpression of *Shh* through *Pax2-cre*-driven expression of *Smo*, which encodes a G protein-coupled receptor, results in exaggerated development of both the IC and the cerebellum [55]. The expression of *Isl1* under *Pax2* regulatory sequences causes a modest reduction in the size of the IC and partially disrupts its normal function [56]. Downstream of *Pax2* are *Pax3, Pax7,* and *Meis2*, which influence the development of the IC roof plate [57]. The expression of *Otx2* is driven by *Sox2, Ascl1, Neurod1, Neurog2*, and *Dll3*, shaping the early initiation and differentiation of the IC [58]. The patterning gene *Dbx1* is a crucial factor for the postnatal survival of the IC [54]. Beyond this early regulation, downstream glutamate, glycine, and GABAergic-expressing neurons in the IC require further investigation [19].

The diencephalon belongs to prosomere 2, and gene deletions reveal its dependence on *Shh*. Prosomere 2 is found caudally to the neuropore. It is separated from the zona limitans [59], which divides *Foxg1* in prosomere 3 [60]. MGB development begins with connections from the IC forming between E13 and E18 before the onset of auditory sensory perceptions. *Pax6* is crucial for the forebrain and the thalamus [61]. Early expressed genes include *Wnt3, Tcf4, Meis2,* and *Irx3*, some of which are essential for early development, such as *Zic4* and *Foxp2*. Furthermore, *Foxp2* is a key gene for speech and language development [62]. *Tbr1* is expressed early and defines glutamatergic neurons in the forebrain. The connections of these neurons are partially affected by the targeted deletions of *Pax6, Foxp2, Wnt3, Tcf4,* and *Irx3*. Several downstream genes have been shown, including multiple *bHLH* genes (*Neurog2, Ascl1, Olig2, and Neurod1*). More analyses of conditional deletions are required to clarify the gene interactions within the thalamus [63].

The developing forebrain, including the auditory cortex (AC), is regulated by key transcription factors such as *Otx1, Pax6, Shh, Emx2,* and *Foxg1* [61]. Additionally, a set of genes is necessary for the development of a normal forebrain, including *Dlx, Nkx2.1, Tbx1, and Tbx2*. Transcription factors vary in the developing forebrain in a precise, inside-out manner, establishing reproducible spatiotemporal patterns of gene regulatory networks (Figure 3). For example, *Pax6* deletion alters the expression of genes such as *Ascl1, Neurog2, Neurod1, Tbr1, Sox5, Sox9*, and *Hes5*, while other genes like *Foxg1* remain largely unaffected [61]. An opposing interaction between *Pax6* and *Foxg1*, both of which depend on *Shh* and *BMPs,* regulates the competence of cortical cells. A double loss of *Pax6* and *Foxg1* abolishes *Ascl1, Olig2, Gsx2, and Dlx1/2/3*, which are redirected to a distinct neuronal variant. *Neurog2* acts downstream to regulate *Neurod1* expression, which is critical for the differentiation of hippocampal granule neurons [63].

In mice, AC neurons are generated between E11.5 and E13.5, with the earliest-born neurons being Cajal-Retzius neurons that develop slightly earlier, between E10.5 and E12.5. Neuronal migration plays a crucial role in the development of distinct cortical layers [64]. The early cortical structure, known as the preplate differentiates into two main regions: the marginal zone and the subplate zone [65]. Subplate neurons are the first to receive input from the thalamus, which is later redirected to cortical layer 4 in the adult brain. The maturation of inputs from cortical neurons shows a developmental delay in the AC, which undergoes functional reorganizations to form orientation columns. Initially, cortical layer 4 is innervated indirectly via the subplate, while direct thalamic input follows as development progresses. Delayed innervation of cortical layer 4 occurs after a first innervation of the subplate neurons. While most subplate neurons are lost in adults, a subset persists into adulthood within layer 6 [66]. Surviving subplate neurons may continue to influence altered or compensatory circuits. Disruptions in the precise timing, migration, or survival of AC neurons can cause neurological disorders [64]. Various activations primarily involve glutamatergic inputs to the subplate and layer 4, which receive both AMPA and NMDA receptor-mediated inputs, and local GABAergic neuronal interneurons also contribute to early inhibitory circuits. A progressive segregation is established in a discrete input/output relationship between the MGB and the AC. Further research is needed to fully map the reciprocal connections from the AC back to the MGB, midbrain, and brainstem, which likely contribute to feedback modulations and auditory plasticity [67].

## Conclusions

This review provides an overview of the key genes required for the early development of the cochlea, brainstem, midbrain, and forebrain. Critical genes, such as *Neurog1*, which is essential for neuronal development, and *Atoh1*, which is required for hair cell and dorsal brainstem development, are indispensable. The deletion of these genes results not only in the absence of the cochlea and brainstem but may also affect the development of the midbrain and forebrain. Early cochlear fiber projections to the brainstem are crucial for establishing tonotopic organization, which refers to the spatial arrangement of sound frequency processing. Spiral ganglion neurons play a pivotal role in transmitting auditory signals from hair cells to the cochlear nuclei. However, bilateral projections from the cochlear nuclei form the basis for binaural hearing, allowing for the perception of sound localization and auditory integration that interacts with the cochlear nuclei, the superior olivary complex, the lateral lemniscus, and the inferior colliculi. This bilateral input continues to the forebrain and auditory cortex, which are crucial for complex auditory processing. We briefly present genetic mutations that disrupt these auditory pathways and discuss their potential implications for the development of auditory system processing. The forebrain, without input from the cochlear nuclei, requires a novel approach to selectively delete *Neurog1* and/or *Atoh1* that do not survive long enough to investigate the loss of this input on forebrain development.
